# Subcutaneous infliximab in Crohn’s disease patients with previous immunogenic failure of intravenous infliximab

**DOI:** 10.1007/s00384-024-04727-3

**Published:** 2024-09-25

**Authors:** Julia Husman, Karin Černá, Katja Matthes, Maximilian Gilger, Maia Arsova, Alexandra Schmidt, Nadia Winzer, Anna-Magdalena Brosch, Franz Brinkmann, Jochen Hampe, Sebastian Zeissig, Milan Lukáš, Renate Schmelz

**Affiliations:** 1 Department of Medicine 1, University Hospital Carl Gustav Carus Dresden, Technische Universität (TU), Fetscherstrasse 74, 01307 Dresden, Germany; 2 Clinical and Research Center for Inflammatory Bowel Disease ISCARE and First Faculty of Medicine, Charles University, Prague, Czech Republic; 3 Center for Regenerative Therapies Dresden (CRTD), Technische Universität (TU), Dresden, Germany; 4 Department of Internal Medicine A, University Medicine Greifswald, Greifswald, Germany

**Keywords:** Subcutaneous infliximab, Immunogenic failure, CT-P13, Refractory Crohn’s disease

## Abstract

**Purpose:**

Immunogenicity is a major reason for secondary loss of response to infliximab (IFX). Recent work suggested potentially lower immunogenicity of subcutaneous (SC) compared to intravenous (IV) IFX. However, it is unknown whether re-exposure to IFX SC after secondary loss of response and immunogenicity to its intravenous formulation is safe and effective.

**Methods:**

In a retrospective cohort study conducted at two medical centers, patients with clinically (Harvey-Bradshaw Index ≥ 5) and/or biochemically (fecal calprotectin > 250 µg/g) active Crohn’s disease (CD) and previous immunogenic failure of IFX IV underwent exposure to IFX SC. Harvey-Bradshaw Index, fecal calprotectin, IFX serum concentration, and anti-drug antibodies were assessed until month 12.

**Results:**

Twenty CD patients were included. The majority of patients (90%) had previous treatment with three or more biologics. Fifteen (75%) and ten (50%) of 20 patients continued IFX SC treatment until months 6 and 12, respectively. No immediate hypersensitivity reactions were observed. Two patients discontinued IFX SC treatment because of delayed hypersensitivity at week 2 and week 4. IFX serum concentrations increased from baseline to month 12, while anti-drug antibody levels decreased. Combined clinical and biochemical remission at month 12 was observed in seven of 20 patients (35%).

**Conclusion:**

Subcutaneous infliximab treatment of Crohn’s disease patients with previous immunogenic failure of intravenous infliximab was well tolerated and effective in a cohort of patients with refractory Crohn’s disease.

**Supplementary Information:**

The online version contains supplementary material available at 10.1007/s00384-024-04727-3.

## Introduction

Infliximab (IFX), a monoclonal antibody directed against tumor necrosis factor (TNF), is a mainstay of treatment for patients with inflammatory bowel disease (IBD) [1–4]. However, long-term efficacy, particularly of intravenous (IV) formulations of IFX, is limited by significant rates of secondary loss of response [5–7]. While secondary loss of response is of multifactorial origin, the immunogenicity of IFX, a chimeric antibody originally raised in mice, significantly contributes to this phenomenon [8]. As such, the PANTS study demonstrated the development of anti-drug antibodies (ADAs) against infliximab in more than 60% of Crohn’s disease (CD) patients by week 54 of treatment [5]. This predicted low IFX concentrations, which in turn predicted treatment failure. Various factors have been suggested to influence the immunogenicity of infliximab including, among others, the genetic background of the patient, drug dosing and intervals, episodic versus scheduled treatment, drug trough levels, or smoking [5, 9–12]. Moreover, previous work has suggested that the route of biologic administration may also influence antigenicity [13, 14]. Recently, a subcutaneous (SC) formulation of the IFX biosimilar CT-P13 has been approved for the treatment of CD and ulcerative colitis (UC), among other immune-mediated diseases [15]. In a phase I study that compared IFX SC and IFX IV treatment in patients with active IBD, higher IFX trough levels were observed in the SC compared to the IV maintenance group [16]. Moreover, albeit not reaching statistical significance, neutralizing anti-IFX ADAs at week 22 were numerically lower in the SC (6%) compared to the IV (15%) maintenance arm in this study. Similar observations were made in rheumatoid arthritis patients in a phase I/III trial: 69.5% of patients were positive for neutralizing anti-IFX ADAs in the SC group vs. 85.6% in the IV group [17]. Another post hoc analysis demonstrated significantly lower immunogenicity in rheumatoid arthritis and CD patients receiving IFX SC compared to IV [18]. In conclusion, these data suggest potentially lower immunogenicity of IFX SC compared with its intravenous formulation [15, 19]. Mechanisms possibly contributing to these observations are that higher IFX trough concentrations associated with SC compared to IFX IV may induce high-zone tolerance and decreased immunogenicity resulting in decreased formation of drug–antibody immune complexes [20]. In light of these findings, we asked the question of whether patients with previous immunogenic failure on IFX IV can be safely re-exposed to IFX SC. In support of this concept, data from patients with immunogenic failure or allergic reactions to IFX IV documented the safety of re-exposure to subcutaneous IFX [21, 22]. However, confirmation of these results in a larger patient population is required. Moreover, data documenting the clinical effectiveness of exposure to IFX SC is needed to confirm the clinical value of such a strategy. Here, we report the effectiveness, safety, and pharmacokinetics of IFX SC in CD patients with immunogenic failure on IFX IV in a retrospective analysis conducted at two medical centers in Germany (Dresden) and the Czech Republic (Prague).

## Materials and methods

### Patients and procedures

This retrospective study was performed at the Dresden University Hospital (TU Dresden, Dresden, Germany), the Clinical and Research Centre for Inflammatory Bowel Disease (ISCARE), and the First Faculty of Medicine (Charles University, Prague, Czech Republic) during the period of February 2022 to August 2023. All patients had clinically or biochemically active CD and a history of immunogenic treatment failure during previous IFX IV therapy (defined as secondary loss of response and high measurable IFX ADA). The median interval between the last IFX infusion and the start of IFX SC treatment was 8 years (range 2–20 years). At the discretion of the treating clinician and multidisciplinary team, IFX SC (CT-P13) treatment was initiated using one of the following regimens: (1) 120 mg IFX SC at weeks 0, 1, 2, and 3, followed by 120 mg IFX SC maintenance therapy every other week (EOW). (2) Direct start with 120 mg IFX SC maintenance therapy EOW without induction. Maintenance treatment for the cohort consisted of 120 mg IFX SC EOW. A subset of patients underwent interval shortening to 120 mg IFX SC every week or dose intensification to 240 mg IFX SC EOW. Harvey-Bradshaw Index (HBI), IFX serum concentrations, IFX ADA, fecal calprotectin (FCP), and C-reactive protein (CRP) were determined at week 0, week 2 (weeks 1–3), month 3 (weeks 12–14), month 6 (weeks 24–30), and month 12 (weeks 48–54). Previous work demonstrated that subcutaneous IFX therapy is associated with stable individual serum levels without *bona fide* drug peaks and troughs [16, 23]. Therapeutic drug monitoring of CT-P13 was therefore performed at the time point of the next scheduled clinic appointment and not necessarily on the day before the next SC injection. Furthermore, we recorded details of dose escalation, interval shortening, adverse events, and discontinuation of infliximab. Clinical remission was defined as HBI < 5. FCP < 250 µg/g was considered biochemical remission. Treatment persistence reflects continuous, uninterrupted maintenance treatment with IFX SC.

### Drug level and anti-drug antibody assay

Free infliximab concentration was measured using the Immundiagnostik AG (Bensheim, Germany) enzyme-linked immunosorbent assays (ELISA) for Infliximab drug level kit (K 9655) or the ImmunoGuide AybayTech Biotechnology kit (Ankara, Türkiye) Infliximab ELISA (IG-AB101) at the Dresden and Prague sites, respectively. Free antibodies against infliximab were measured using either the Immundiagnostik AG (Bensheim, Germany) IDKmonitor® Infliximab free ADA kit (K9650) (cut off of detection for ADA = 10 AU/ml) in Dresden or the i-Tracker Anti-Drug Infliximab kit (CTI-003) from TheraDiag (Croissy Beaubourg, France) (level of detection for ADA, 10–2000 ng/ml) in Praque. As mentioned above, different assays for IFX serum concentration and ADA measurements were used at the Dresden and Prague sites, respectively. However, longitudinal measurements were for all patients consistently performed using the same assay.

### Statistical analysis

Descriptive statistics were used to analyze demographic, disease, and treatment characteristics. Categorical variables were summarized as frequency (%). Continuous variables were summarized as median and interquartile range (IQR) for non-normally distributed data. Kaplan–Meier survival analysis was used to analyze treatment persistence. Descriptive methods were used to analyze reasons for treatment discontinuation. We performed a univariate analysis (Fisher’s exact test for contingency tables, Man-Whitney test for non-normally distributed data) to assess variables associated with clinical remission at month 12. All analyses were carried out using GraphPad Prism (version 9.0.0).

## Results

### Patient cohort

Twenty CD patients were analyzed in this study. Baseline demographic parameters are shown in Table 1. Patients were extensively pretreated (90% had previous treatment with ≥ 3 biologics including IFX IV) (Table 1). All patients showed clinically and/or biochemically active CD before commencing IFX SC. Median HBI at baseline was 5 (4.5, IQR), and median FCP was 1115 µg/g (1722 µg/g, IQR).

**Table 1:** Baseline demographics of the 20 CD patients included

Demographics	
Age, y (median, IQR)	30.5 (11.25)
Disease duration, y (median, IQR)	15.0 (10.3)
Sex, female (*n*, %)	10 (50%)
Detectable IFX ADA before IFX SC (*n*, %)	20 (100%)
Disease characteristics at baseline	
Age at diagnosis (*n*, %)	
A1 (< 16)	10 (50%)
A2 (17–40)	10 (50%)
A3 (> 40)	0
Disease extent (*n*, %)	
Ileal (L1)	0
Colonic (L2)	2 (10%)
Ileo-colonic (L3)	17 (85%)
Upper GI, (L4)	1 (5%)
Behavior classification (*n*, %)	
B1 (non-stricturing, non-penetrating)	0
B2 (stricturing)	8 (40%)
B3 (penetrating)	12 (60%)
Perianal disease	12 (60%)
Clinical indices at baseline	
HBI Score (median, IQR)	5.0 (4.5)
CRP, mg/ml (median, IQR)	14.9 (25.2)
FCP, µg/g (median, IQR)	1115.0 (1722.8)
Biologic pretreatment (*n*, %)	
2 Biologics (incl. IFX IV)	2 (10%)
3 Biologics (incl. IFX IV)	8 (40%)
≥ 4 Biologics (incl. IFX IV)	10 (50%)
Concomitant therapy (*n*, %)	
Corticosteroids (≥ 20 mg/d)	1 (5%)
Corticosteroids (< 20 mg/d)	3 (15%)
Azathioprine/6-MP	3 (15%)
MTX	6 (30%)

*IQR* interquartile range, *ADA* anti-drug antibodies, *IFX IV* infliximab intravenous, *HBI* Harvey-Bradshaw Index, *CRP* C-reactive protein, *FCP* fecal calprotectin, *MTX* methotrexate

### Infliximab dosing and concomitant therapy

At the physician’s discretion, patients were initiated on subcutaneous IFX using one of the following regimens: (1) 17 (85%) patients received 120 mg IFX SC at weeks 0, 1, 2, and 3 as an induction treatment followed by maintenance with 120 mg IFX SC EOW. (2) Three (15%) patients started directly with 120 mg IFX SC EOW treatment without an induction regimen. For maintenance therapy, 15 patients (75%) received 120 mg IFX SC EOW. Five patients (25%) received a dose escalation to 120 mg IFX SC every week or 240 mg IFX SC EOW at the physician’s discretion. Reasons for dose escalation and interval shortening included insufficient clinical responses and persistence of biochemical markers of active disease (four patients) or body weight > 120 kg (one patient). Time points of dose escalation and interval shortening were between months 2 and 4 (Supplementary Fig. 1). At the time point of IFX SC initiation, 9 patients (45%) received concomitant immunomodulatory therapy: 3 patients (15%) received azathioprine, 6 (30%) methotrexate, and 4 (20%) oral corticosteroids. Azathioprine or methotrexate therapy had been initiated at least 3 months prior to the first subcutaneous infliximab dose. The proportion of ADA-positive patients at baseline did not differ between patients with or without concomitant immunomodulatory therapy (7 of 9 vs. 10 of 11 patients, *p* = 0.6). Seven of 9 patients with immunomodulatory therapy at baseline were treated with IFX SC until month 6 or longer. Azathioprine or methotrexate was stopped at month 3 for most of them (6 of 7 patients, 86%). One patient continued methotrexate therapy until month 12, all other patients had discontinued immunomodulatory or steroid therapy (Supplementary Table 1).

### Treatment persistence and safety

**Fig. 1:**
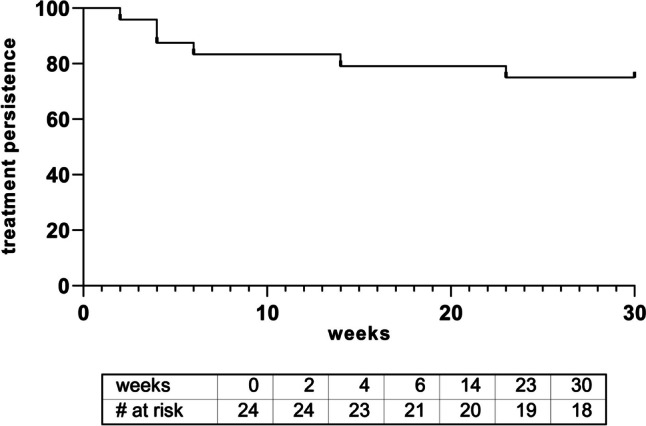
Treatment persistence of IFX SC therapy. Kaplan–Meier curve showing treatment persistence on subcutaneous (SC) infliximab (IFX)

During the follow-up period, treatment persistence was 75% (15/20 patients) and 50% (10/20 patients) at 6 months and 12 months, respectively, with a median treatment duration of 41 weeks (range, 4–52 weeks) (Fig. 1). Reasons for IFX SC discontinuation are shown in Table 2. Lack of effectiveness led to infliximab withdrawal in 7 cases (35%). Two patients (10%) had to stop subcutaneous infliximab because of a delayed hypersensitivity reaction (angioedema, urticaria, and arthralgia) at weeks 2 and 4, respectively. One patient (5%) was lost to follow-up after month 6 due to moving and changing physicians. Furthermore, 1 patient (5%) developed a mild self-limiting injection site reaction, which did not lead to discontinuation of IFX treatment. No immediate hypersensitivity reaction upon subcutaneous infliximab administration was observed.

**Table 2:** Reasons for IFX SC discontinuation before month 12

Reason for IFX SC discontinuation	Number (%)
Worsening of disease activity	7 (35%)
Delayed hypersensitivity reaction	2 (10%)
Lost to follow-up	1 (5%)
Total	10 (50%)

*IFX SC* infliximab subcutaneous

### Infliximab pharmacokinetics and immunogenicity

**Fig. 2:**
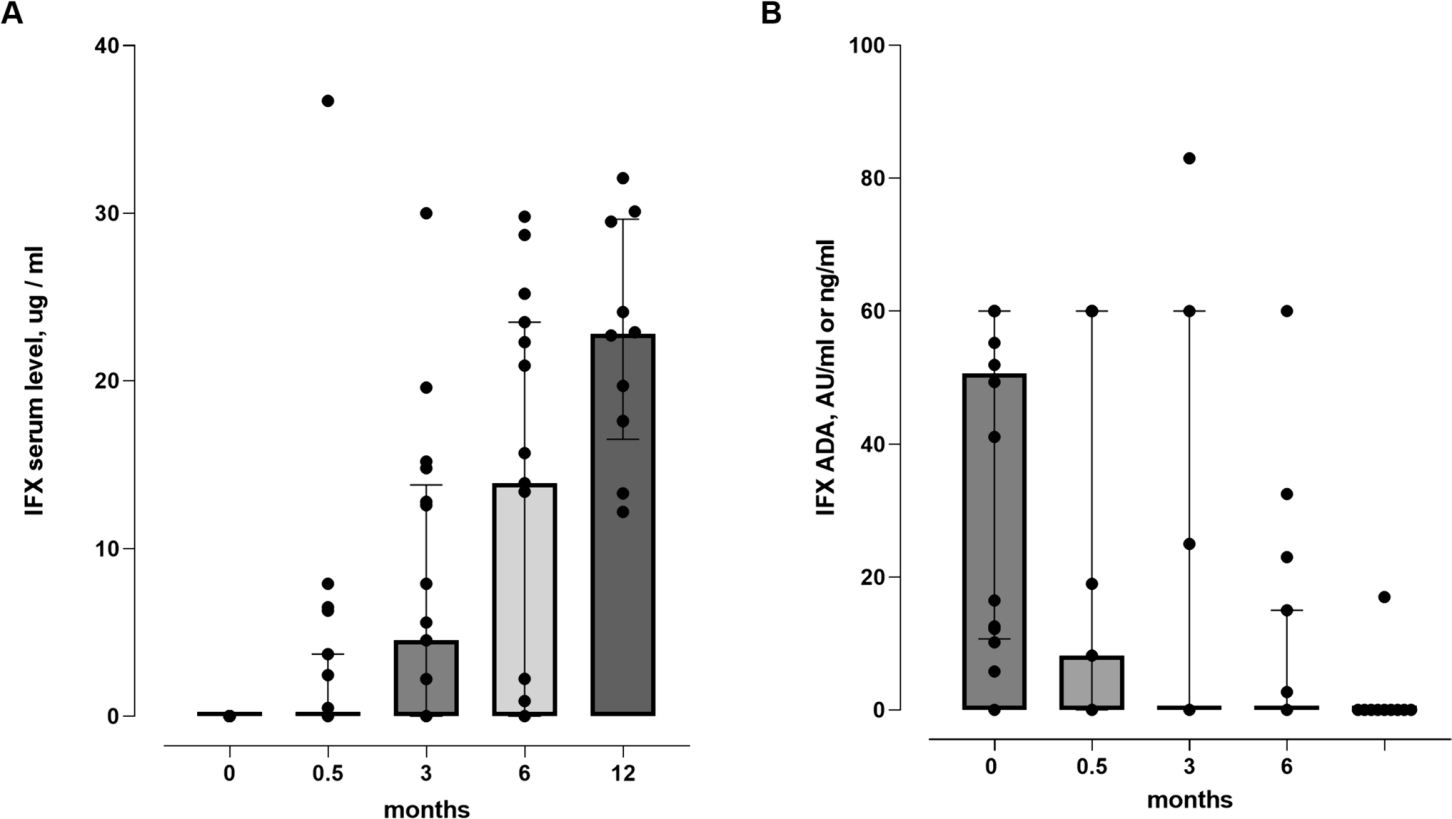
IFX serum concentrations and IFX ADA. Aligned dot plot of **A** individual infliximab (IFX) serum concentrations and **B** IFX anti-drug antibodies (ADA) from baseline to month 12. The horizontal lines represent the median values (bold) with interquartile range

Large inter-individual variations of IFX serum concentrations were observed. Infliximab serum concentrations increased from baseline to month 12 (Fig. 2A). The median IFX concentration at baseline and months 3, 6, and 12 was 0 (0, IQR), 4.5 µl/ml (12.8, IQR), 13.9 µl/ml (22.5, IQR), and 22.8 µl/ml (10.0, IQR), respectively. The highest IFX serum concentrations were reached in patients who had received weekly injections of 120 mg IFX SC at weeks 0, 1, 2, and 3 as induction therapy (Supplementary Fig. 2). All patients had a history of detectable antibodies to infliximab before commencing IFX SC treatment. IFX ADAs were detectable in 17 of 20 patients at baseline (85%), 5 of 15 remaining patients (33.3%) at month 6, and 1 of 10 remaining patients (10%) at month 12. The median IFX ADA value at baseline and months 3, 6, and 12 was 50.6 AU/ml (48.3, IQR), 0.0 AU/ml (60.0, IQR), 0.0 AU/ml (8.9, IQR), and 0.0 AU/ml (0, IQR), respectively (Fig. 2B).

### Clinical effectiveness and biomarker responses

**Fig. 3:**
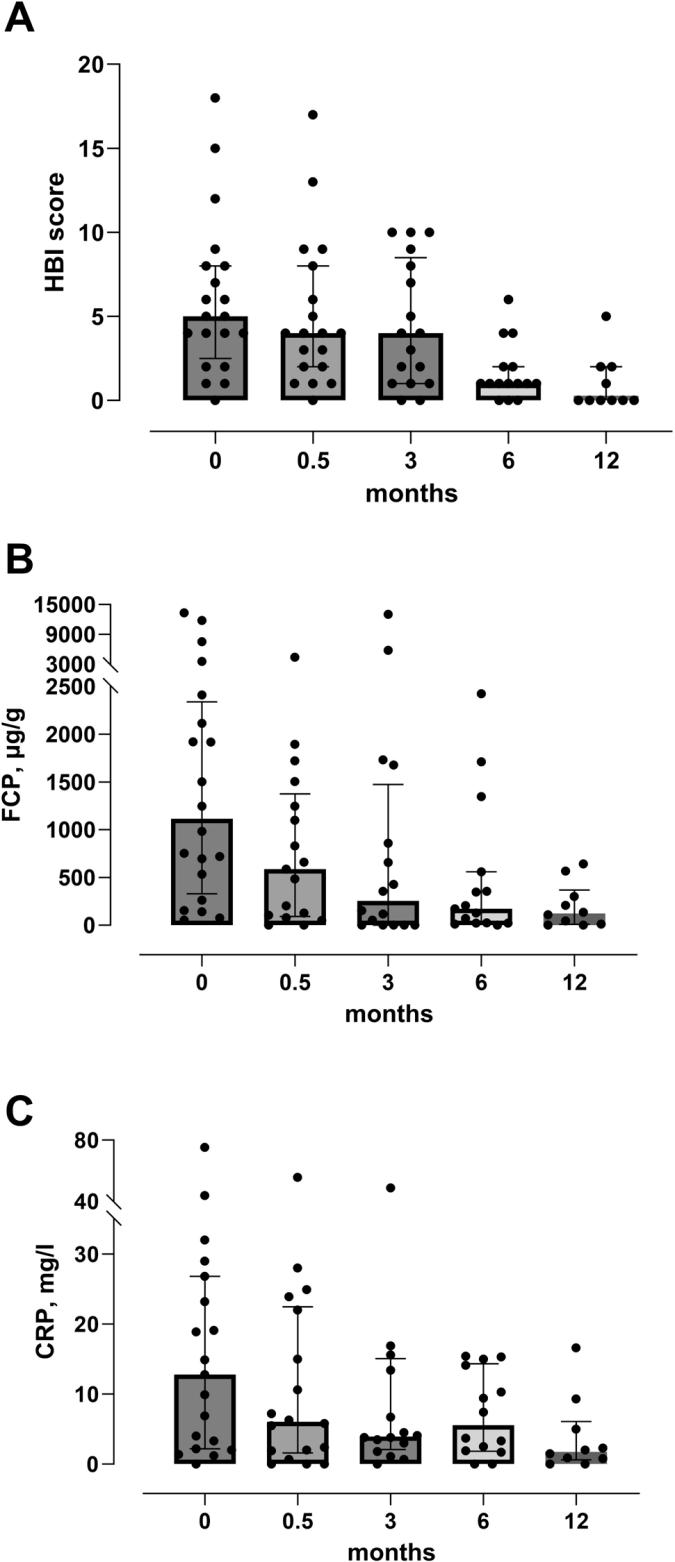
HBI, FCP, and CRP responses during IFX SC treatment. A Harvey-Bradshaw Index (HBI), B fecal calprotectin (FCP), and C C-reactive protein (CRP) are shown in an aligned dot plot for all patients from baseline to month 12

Combined clinical and biochemical remission at month 12 was observed in 7 of 20 patients (35%). Trends in biomarker responses (CRP, FCP) are shown in Fig. 3. Median FCP values at baseline and months 3, 6, and 12 were 1115 µg/g (1722, IQR), 254 µg/g (1064, IQR), 173 µg/g (457, IQR), and 124 µg/g (257, IQR), respectively. We performed univariate analysis to assess the effect of patient, clinical, or biochemical parameters on clinical remission at month 12. Variables included age, sex, FCP, CRP, HBI, and concomitant immunomodulatory therapy at baseline as well as infliximab serum levels and IFX ADA concentrations. IFX serum level > 3 µg/ml during follow-up was the only parameter significantly associated with clinical remission at month 12 (Table 3). Furthermore, ROC analysis failed to detect a threshold of IFX ADA levels associated with the failure of IFX SC (Supplementary Fig. 3).

**Table 3:** Effect of clinical variables on clinical remission in month 12

Factor		Clinical remission month 12		*P* value
		Yes (*n* = 9)	No (*n* = 11)	Univariate
Age	y, median (IQR)	27 (12)	33 (10)	0.18
Sex	Female, *n* (%)	4 (44)	6 (55)	> 0.99
	Male, *n* (%)	5 (66)	5 (45)	
FCP at baseline	µg/g, median (IQR)	1451 (3224)	1247 (3445)	0.72
CRP at baseline	mg/l, median (IQR)	9.9 (23.2)	18.9 (30.0)	0.65
HBI at baseline	Score, median (IQR)	4 (9)	6 (4)	0.25
Concomitant therapy AZA/MTX at baseline	Yes, *n* (%)	5 (66)	4 (36)	0.65
	No, *n* (%)	4 (44)	7 (64)	
IFX Serum level	> 3 µg/ml, *n* (%)	9 (100)	3 (27)	**0.001**
	< 3 µg/ml, *n* (%)	0	8 (73)	
IFX ADA	AU/ml or ng/ml, median (IQR)	41.0 (52)	51.9 (47.8)	0.66

*IQR* interquartile range; *FCP* fecal calprotectin; *CRP* C-reactive protein; *HBI* Harvey-Bradshaw Index; *AZA* azathioprine; *MTX* methotrexate; *IFX* infliximab

## Discussion

Previous work has suggested potentially lower immunogenicity of IFX SC compared to IFX IV [15–20]. These findings may influence the physician’s choices regarding the route of IFX administration but also raise the question of whether patients who had previously experienced secondary loss of response due to immunogenicity on IFX IV can be safely and effectively re-exposed to IFX SC. Here, we demonstrate in a cohort of extensively pretreated CD patients that exposure to subcutaneous CT-P13 after immunogenic failure on IFX IV is safe and not associated with severe systemic immune responses. Furthermore, we document evidence of the effectiveness of IFX SC treatment in these patients with difficult-to-treat CD.

The presence of IFX ADA is associated with a significantly higher risk of acute IFX-associated infusion reactions, but not delayed hypersensitivity reactions in IBD patients [24]. In a recently published case series of 4 patients with previous immunogenic failure of IFX IV, Caron et al. demonstrated the absence of severe systemic immune responses upon exposure to subcutaneous CT-P13 [21]. In a larger cohort based on 20 CD patients, we now confirm the absence of severe, systemic immune-mediated responses upon subsequent exposure to IFX SC. We observed two cases of delayed hypersensitivity (10% of patients). Additionally, 1 patient (5%) developed a mild, self-limiting injection site reaction. These data correspond well to previously reported rates of delayed hypersensitivity (3% of patients in Schreiber et al.) and injection site reactions (28% in Schreiber et al. and 3% in Smith et al.) in populations without previous immunogenic failure on IFX IV [16, 25]. History of secondary loss of response and immunogenicity to IFX IV in our cohort was therefore not associated with a considerable increase in immune-related adverse events.

An intriguing observation of our study was that re-exposure to IFX via subcutaneous administration was associated with a continuous decrease in ADA serum concentrations. These results are in line with the previous phase I trial by Schreiber et al. which demonstrated that a switch from IFX IV to IFX SC maintenance was associated with a numeric decrease in the percentage of patients positive for neutralizing and total ADA [16]. High-zone immune tolerance and decreased formation of drug–antigen immune complexes due to higher IFX trough concentrations may contribute to these phenomena [20].

Throughout the 12-month observation period, IFX serum concentrations continuously increased. The highest IFX serum levels were observed in patients with weekly IFX SC induction for 4 weeks, an induction scheme closely related to that approved for rheumatoid arthritis [26]. Median IFX serum concentration at month 6 was 13.9 µg/ml (22.5, IQR), which was slightly lower than the observed mean trough level of 20.2 µg/ml reported at week 24 in the phase I trial of Schreiber et al. In addition, IFX serum levels rose substantially slower compared to the formerly mentioned study with IV induction and indeed had not reached a plateau by month 6 [16]. Interestingly, two patients of our cohort (10%) developed significant IFX serum levels only after month 6. As such, IFX SC induction, performed with the rationale of avoiding systemic immune responses potentially observed with IFX IV administration, might be associated with a slower rise in IFX serum levels compared to IV induction. Extended periods of treatment or intensified dosages may therefore be required to observe full clinical efficacy.

Our data suggest that exposure to IFX SC in patients with immunogenic failure on IFX IV is associated with clinical effectiveness in a substantial proportion of patients, even in the context of extensive biologic pretreatment. Combined clinical and biochemical remission at month 12 was observed in 7 of 20 patients (35%).

Our study and the conclusions derived from this work have several limitations: The study was of modest group size, and only CD patients were included, which is a limiting factor for the generalizability of our findings. Furthermore, our data does not exclude the possibility of rare occurrences of severe systemic immune responses to IFX SC exposure in a larger cohort of patients with immunogenic IFX failure. In addition, while the current label of CT-P13 SC mandates intravenous IFX induction for CD and UC patients, all patients in our study received direct induction with CT-P13 SC based on safety concerns. The conclusions derived from our cohort therefore mainly apply to patients with direct CT-P13 SC treatment. Differing IFX SC dosing schemes may introduce variability to our results. Most patients (17 of 20 patients) received an induction scheme consisting of four times 120 mg IFX SC weekly, while three patients commenced IFX SC treatment with the maintenance dose of 120 mg IFX EOW directly. In addition, five of our 20 patients received a dose escalation. Patients receiving the subcutaneous induction scheme had evidently higher infliximab serum concentrations than patients without as shown in the supplementary material. Last, the assays used for measuring antibodies to infliximab were drug-sensitive and differed between the two medical sites, which limits the ability to compare results between centers.

The continuously expanding spectrum of biologic and small molecule therapies for patients with CD offers various therapeutic options to patients with immunogenic failure of IFX. However, as illustrated by the patient cohort studied here, a subset of individuals fails subsequent therapies resulting in treatment-refractory active disease that may warrant re-exposure to IFX as a rescue option. Furthermore, areas such as fistulizing CD, pregnancy, breastfeeding, and comorbidities can provide clear indications for anti-TNF therapies. The assessment of the efficacy and safety of IFX re-exposure in IBD patients with previous immunogenic failure to IFX IV will benefit from confirmation in a prospective, multi-center trial that involves ultrasound and/or endoscopy as objective endpoints. Furthermore, future work is warranted to identify the optimal IFX induction scheme for patients with previous immunogenic IFX failure.

## Supplementary Information

Below is the link to the electronic supplementary material.

Supplementary file1 (PDF 527 KB)

## Data Availability

The data underlying this article will be shared on reasonable request to the corresponding author.
