# Supplementary material for: Subcutaneous infliximab in Crohn’s disease patients with previous immunogenic failure of intravenous infliximab
Source: Int J Colorectal Dis. 2024 Sep 25;39(1):151. doi: 10.1007/s00384-024-04727-3 (PMC11422436; doi:10.1007/s00384-024-04727-3)
Supplement: Supplementary file 1 — Supplementary file1 (PDF 527 KB) [file 384_2024_4727_MOESM1_ESM.pdf]

Supplementary Data

Subcutaneous infliximab in Crohn's disease patients with previous immunogenic failure of intravenous infliximab

International Journal of Colorectal Disease

Authors: Julia Husman, MD*[1]; Karin Černá, MD*[2]; Katja Matthes, MD*[1]; Maximilian Gilger, MD[1]; Maia Arsova, MD[1]; Alexandra Schmidt, MD[1]; Nadia Winzer, MD[1]; Anna-Magdalena Brosch, MD[1]; Franz Brinkmann, MD[1]; Jochen Hampe, MD, Professor[1]; Sebastian Zeissig, MD, Professor [1,3,4]; Milan Lukáš, MD, Professor*[2]; Renate Schmelz, MD*[1]
** Authors contributed equally to the manuscript in the role ascribed to first and last authors.*

[1] Department of Medicine 1, University Hospital Carl Gustav Carus Dresden, Technische Universität (TU) Dresden, Germany
[2] Clinical and Research Center for Inflammatory Bowel Disease ISCARE and First Faculty of Medicine, Charles University, Prague, Czech Republic
[3] Center for Regenerative Therapies Dresden (CRTD), Technische Universität (TU) Dresden, Germany
[4] Department of Internal Medicine A, University Medicine Greifswald, Greifswald, Germany

Corresponding Author: Dr. med. Renate Schmelz, Department of Medicine 1, University Hospital Carl Gustav Carus Dresden, TU Dresden, Fetscherstrasse 74, 01307 Dresden, Germany. Email: renate.schmelz@ukdd.de

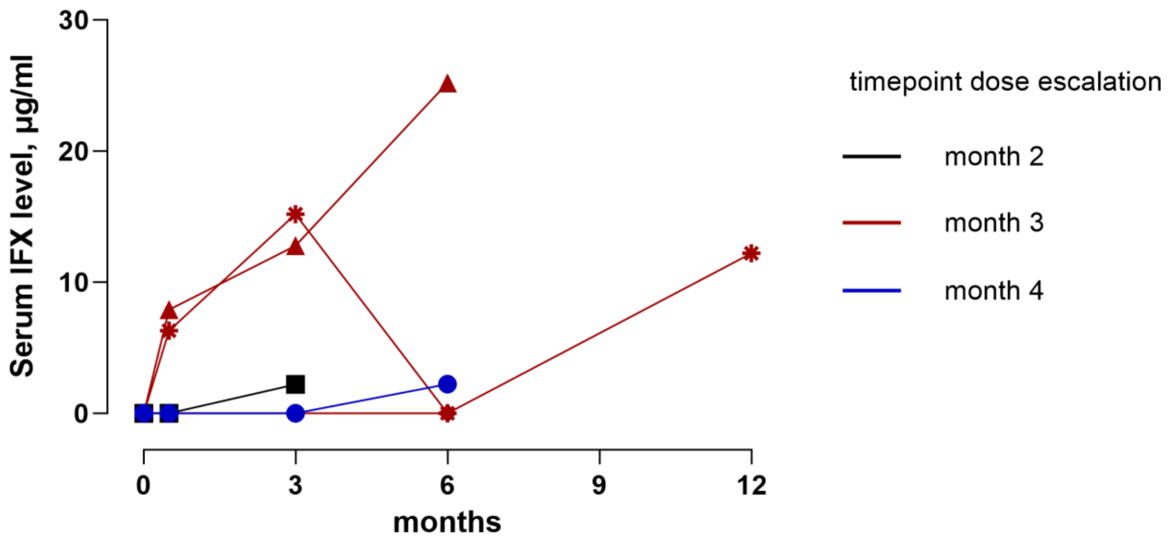

Supplementary Figure 1: Individual development of IFX serum concentration in patients with IFX SC interval shortening or dose escalation.

Of five patients who received an IFX dose intensification (IFX SC 240 mg EOW) or interval shortening (IFX SC 120 mg weekly), four (80%) continued IFX SC treatment until month 6 and 1 (20%) until month 12. Dose intensification was started between months 2 and 4.

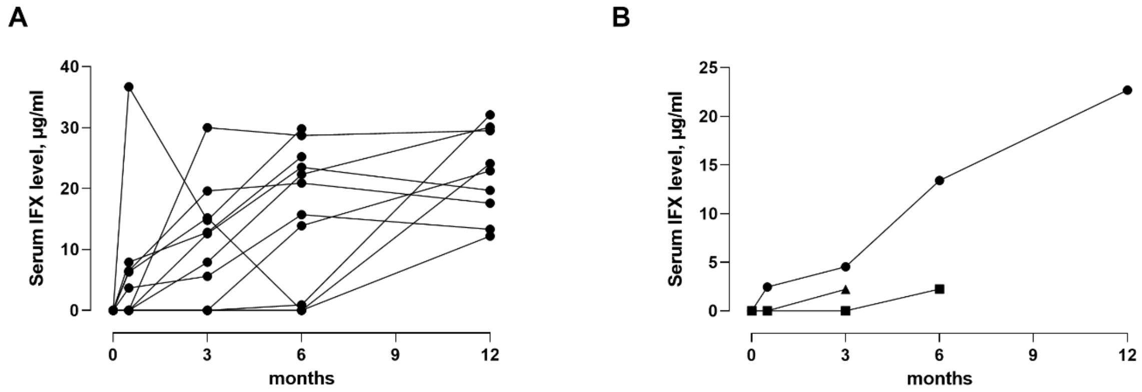

Supplementary Figure 2: Individual development of IFX serum levels stratified by induction regimen. Individual trends in IFX serum concentrations for patients who (A) received an induction with four IFX SC 120 mg weekly injections (n = 17), (B) were started directly on 120 mg IFX SC EOW (n = 3).

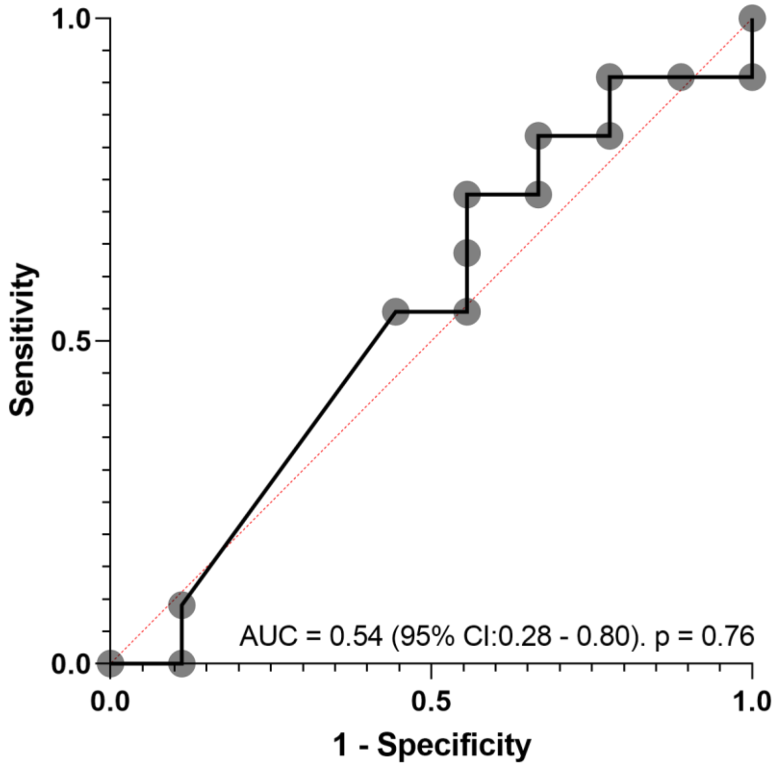

Supplementary Figure 3: ROC analysis of IFX ADA levels associated with failure of IFX SC. ROC analysis for infliximab anti-drug antibodies levels at baseline as a predictor of achieving clinical remission at month 12. AUC, area under the curve.

Supplementary Table 1. Concomitant therapy at baseline, month 3, 6 and 12

	**Baseline (n=20)**	**month 3 (n=17)**	**month 6 (n=15)**	**month 12 (n=10)**
Corticosteroids (≥20mg/d)	1 (5 %)	1 (6 %)	0	0
Corticosteroids (<20mg/d)	4 (20 %)	0	0	0
Azathioprine	3 (15 %)	0	0	0
Methotrexate	6 (30 %)	2 (12 %)	1 (7 %)	1 (10%)
**Total**	**14 (70 %)**	**3 (18 %)**	**1 (7 %)**	**1 (10 %)**
